# Determinants of Tree Assemblage Composition at the Mesoscale within a Subtropical Eucalypt Forest

**DOI:** 10.1371/journal.pone.0114994

**Published:** 2014-12-12

**Authors:** Jean-Marc Hero, Sarah A. Butler, Gregory W. Lollback, James G. Castley

**Affiliations:** Environmental Futures Research Institute, School of Environment, Griffith University, Gold Coast, Qld 4222, Australia; University of New South Wales, Australia

## Abstract

A variety of environmental processes, including topography, edaphic and disturbance factors can influence vegetation composition. The relative influence of these patterns has been known to vary with scale, however, few studies have focused on environmental drivers of composition at the mesoscale. This study examined the relative importance of topography, catchment flow and soil in influencing tree assemblages in Karawatha Forest Park; a South-East Queensland subtropical eucalypt forest embedded in an urban matrix that is part of the Terrestrial Ecosystem Research Network South-East Queensland Peri-urban SuperSite. Thirty-three LTER plots were surveyed at the mesoscale (909 ha), where all woody stems ≥1.3 m high rooted within plots were sampled. Vegetation was divided into three cohorts: small (≥1–10 cm DBH), intermediate (≥10–30 cm DBH), and large (≥30 cm DBH). Plot slope, aspect, elevation, catchment area and location and soil chemistry and structure were also measured. Ordinations and smooth surface modelling were used to determine drivers of vegetation assemblage in each cohort. Vegetation composition was highly variable among plots at the mesoscale (plots systematically placed at 500 m intervals). Elevation was strongly related to woody vegetation composition across all cohorts (*R^2^*: 0.69–0.75). Other topographic variables that explained a substantial amount of variation in composition were catchment area (*R^2^*: 0.43–0.45) and slope (*R^2^*: 0.23–0.61). Soil chemistry (*R^2^*: 0.09–0.75) was also associated with woody vegetation composition. While species composition differed substantially between cohorts, the environmental variables explaining composition did not. These results demonstrate the overriding importance of elevation and other topographic features in discriminating tree assemblage patterns irrespective of tree size. The importance of soil characteristics to tree assemblages was also influenced by topography, where ridge top sites were typically drier and had lower soil nutrient levels than riparian areas.

## Introduction

A number of environmental processes influence vegetation patterns, which is typically observed as variation in vegetation structure and composition as a continuum along environmental gradients [1]. The relative influence of environmental processes however, varies at different spatial and temporal scales [2], [3], [4]. For example, it is widely accepted that regional vegetation patterns (10–1000 km^2^) are determined by regional variation in climate, landform and soil type [2], [5], [6], [7], [8], [9], [10], whereas local variation (<1 km^2^) is determined by finer scales of soil chemistry, disturbance and biological interactions [11], [12], [13], [14]. What is less clear is how the role of these processes changes from local to regional scales due to few studies being undertaken at mesoscales (a study area of 1–10 km^2^ size with adequate coverage of replicates, [16], [17]).

The relative importance of environmental factors in influencing vegetation patterns has had some attention, with most research focusing on the associations of individual or functional groups of factors such as soil chemistry [18] or topography [19], [20]. These studies typically demonstrate highly significant correlations between species patterns and environmental factors, although correlations at the assemblage level are often relatively low. The few studies that have evaluated a range of factors have demonstrated that individual factors alone do not explain the observed spatio-temporal patterns of vegetation [16]. Consequently vegetation patterns within a landscape may therefore be driven by a number of environmental processes.

The importance of environmental factors on eucalypt community and species levels patterns has also been widely documented, although most studies have occurred at regional [21], [22], [23], [24], [25] or local scales [12], [26], [27]. For example, variations in eucalypt savannas at the regional scale are attributable to soil type gradients [21], [22], [23] or very broad elevation gradients [28], [29]. Similarly, local scale variation in subtropical eucalypt forests varies significantly between valley-floors and ridge-tops [27], which is attributable to hydrological and soil nutrient gradients within a catchment.

The strong associations between vegetation patterns and the environment may also result in structural and compositional differences in size cohorts within an assemblage. This is evident in the marked differences in the determinants of the potential and observed distribution and abundance patterns of seedlings and mature sized vegetation [30]. For example, the potential distribution and abundance of seedlings is codetermined by seed dispersal patterns and seed output, which are in turn influenced by environmental factors and biological interactions, as well as genotype specific effects of individual seeds [31]. In contrast, determinants of realised abundance patterns of seedlings and subsequent abundance within size classes are influenced by a suite of environmental conditions [30], [31]. The differences between the environmental determinants of trees of different size cohorts however have been little studied, with most studies focusing on diameter density curves and predicting population trends [30], [32], [33], [34] rather than on determinants of recruitment variability between size cohorts at the assemblage level.

This study evaluates the role of a range of environmental factors in influencing the variation in tree assemblages across a complex subtropical eucalypt forest mosaic at the mesoscale in eastern Australia. At the mesoscale, the importance of a number of habitat variables including topography, available runoff and soil chemistry and structure may be responsible for determining vegetation patterns among size cohorts. This will be tested independently for trees within three size cohorts at the assemblage level. The study of these relationships will provide new insight into the relative importance of environmental determinants of assemblages at a scale and in a forest type that has received little attention in the scientific literature.

## Materials and Methods

### Site Description

**Figure 1:**
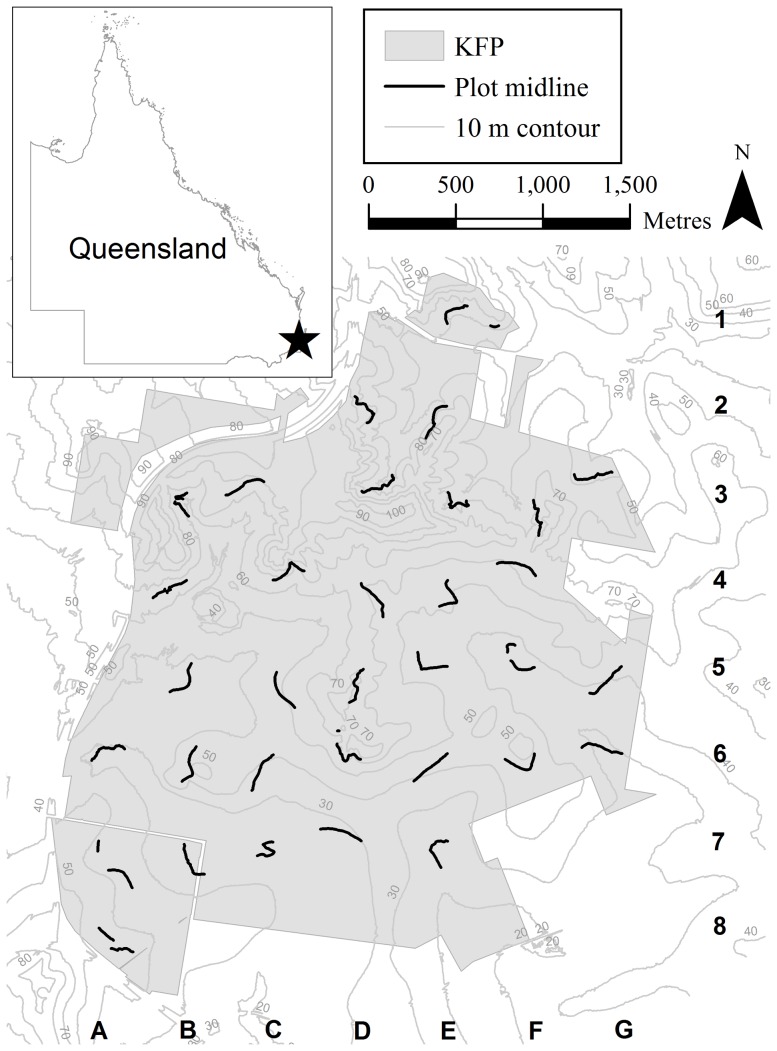
Plot layout within Karawatha Forest Park (KFP) within the TERN SEQ Peri-urban SuperSite. The midlines (thick black lines), and 10 m contour lines (thin grey lines with values in metres) demonstrate the positions for thirty three PPBio LTER plots surveyed in this study. The inset shows the location of KFP (star) within Queensland, Australia. Each midline starting point was placed systematically on a grid. These grid locations are also displayed as a combination of a letter and number.

This study was conducted at Karawatha Forest Park (KFP) in southeast Queensland, Australia (Fig. 1), which is located 18 km south of Brisbane (Latitude −27.632 and Longitude 153.084). It is a long-term ecological research site (LTER node) that is part of the South-east Queensland Peri-urban Supersite within the Terrestrial Ecosystem Research Network (TERN). More information about TERN Supersites can be found at http://www.tern-supersites.net.au. The forest park covers an area of approximately 900 ha and is characterised by remnant mixed eucalypt forest with a predominately grassy understorey and melaleuca forest with a fern and herbaceous understorey. It is one of the largest remaining peri-urban forests under governance of the Brisbane City Council, with one of the highest richness of eucalypt species within the greater Brisbane region [35]. The vegetation communities are divided into two major groups including wetlands and mosaic mixed eucalypt communities, with the wetlands restricted to areas along the creek systems and dominated by *Melaleuca quinquenervia*. In contrast, the well-drained and infertile sandstone ridgelines in the north of Karawatha are dominated by eucalypt communities [35].

## Experimental Design

Fieldwork was conducted between February and July 2007. Permits were not needed for this study and permission to perform vegetation and soil sampling within KFP was granted by Brisbane City Council (BCC), which governs KFP. The BCC is the single local government authority responsible for the administration of the whole of the metropolitan area of Brisbane. No destructive sampling was undertaken during this study. Sampling at 33 permanent plots followed the Planned Program for Biodiversity Studies (PPBio) protocols developed by Magnusson et al. [36] and described by Hero et al. [37] and Magnusson et al. [38]. The plot locations were positioned using a systematic grid system with dimensions of approximately 3.5 km by 3 km comprised of 8 east-west and 6 north-south gridlines. Grid placement was randomly determined by desktop mapping to maximise the number of plots within the park boundaries. The positioning of plots between gridlines maximised the distance between plots, such that each plot begins approximately 500 m from neighbouring plots. Plot coordinates were determined from desktop mapping and were located on site using a GPS (Fig. 1).

Each plot is 250 m long and 42 m wide with a 2 m wide midline dividing the plot into two 20 m wide sections, with a total sample area of 1 ha. Each 1-ha plot follows the topographic contours thereby minimising within plot variation and maximising between plot variation in terms of catchment area, soil types, topography, and vegetation structure and composition facilitating robust comparison between plots [16], [37], [39]. The plots were permanently established using surveying equipment with metal pegs spaced along the plot midline at 10 m intervals to allow for long-term research and ecological monitoring as part of PPBio in Australia.

A hierarchical spatial design was used to sample woody vegetation ≥1.3 m in height from different size cohorts: trees ≥1–10 cm DBH were sampled within 0.1 ha (4 m×250 m); trees ≥10–30 cm DBH were sampled within 0.5 ha (20 m×250 m), and woody vegetation ≥30 cm DBH were sampled within 1 ha (40 m×250 m). The size classes used in this study are designed to adequately estimate the density of woody species of different sizes that vary in their horizontal spatial density (*sensu* Costa et al. [16]). For example, saplings often present at higher densities than larger trees due to their relatively higher recruitment rates [30], [32] and therefore require a relatively smaller area to estimate densities.

The hierarchical sampling design was used to tag and measure the DBH of all trees rooted within plots. Individuals within Myrtaceae, Proteaceae, Fabaceae, Rhamnaceae and Casuarinaceae were identified to species level where possible, including those from the genera *Acacia*, *Allocasuarina*, *Alphitonia*, *Angophora*, *Banksia*, *Corymbia*, *Callistemon*, *Eucalyptus*, *Melaleuca*, *Lophostemon* and *Persoonia* using taxonomic classification by the Queensland Herbarium [40] for eucalypts and Flora of Australia [41], the Australian Biological Resources Study [42] and the Australian Biological Resources Study [43] for all other species. The abundance of species was collated within each size class for each plot.

The environmental variables measured at each plot included plot topography, soil texture and chemistry. Topographical measurements included altitude, slope, aspect and available runoff was represented by catchment area. Plot altitude was measured from topographical maps, aspect was measured using a compass, and slope was measured using clinometers over a distance of 2 m in the direction of the greatest slope with measures taken every 50 m and averaged for each plot. Catchment area was measured using topographic maps, with catchment area digitized using Autocad 2006 [44] and measured in square metres.

Soil samples within each plot were taken to a depth of 10 cm below the surface of soil at six points (0, 50, 100, 150, 200 and 250 m) along the plot midline and assembled to produce a composite sample for each plot. Prior to analysis, the composite soil samples were dried at room temperature, cleaned of stones and fine roots, and passed through a 2 mm sieve according to the protocol outlined by Kalra and Maynard [45]. Soil particle size distribution (% silt, clay and sand) was determined using the hydrometer method (*sensu* Kalra and Maynard [45]). Soil chemistry analyses included pH, Electrical Conductivity (EC), total carbon (C), total nitrogen (N), total phosphorus (P), Cation Exchange Capacity (CEC), magnesium (Mg^2+^), potassium (K^+^), sodium (Na^+^), calcium (Ca^2+^), nitrates (NO_3_^−^) and nitrites (NO_2_^+^) using standard methods described by Rayment and Higginson [46].

## Analyses

Trees were split into the size cohorts of 1–10 cm DBH, 10–30 cm DBH and>30 cm DBH for all analyses. This is because different selection pressures may affect each size class [31]. All analyses involved a measure of ecological resemblance and we chose to use Gower's similarity coefficient (equation 7.20 in Legendre and Legendre [47]), because it is symmetrical and accounts for the ecological significance of the absence of a species in two or more sites.

The spatial arrangement and autocorrelation of species composition was evaluated using a permutated (*n* = 999) Mantel statistic. The statistic was calculated for distance classes that reflected the spatial arrangement of PPBio plots. A significance test (*P*<0.05) indicates either spatial autocorrelation with positive Mantel statistic values, or dispersion with negative statistic values [47].

The relationship between tree species composition and the environment was examined using indirect gradient analysis. If two explanatory variables had a correlation coefficient>0.7, one of the variables was not included in the analysis [48]. Because of the many soil variables (*n* = 14), a Principal Components Analysis was used to produce major axes from the soil data. The major axes scores were then used as predictor variables. Species composition data were first analysed using a non-metric multidimensional scaling analysis (NMDS). The number of axes used was decided by plotting the stress value against the number of axes. The number of axes was chosen when the reduction of stress stopped decreasing noticeably with increasing axes. The data were centred, rotated and scaled for the NMDS analysis. Smooth surfaces of the explanatory variables were then fitted to the NMDS ordination. The strength of the relationship between the ordination and the fitted environmental vectors is reflected in an *R^2^* coefficient of determination. All analyses were carried out in the R program [49] using the package vegan [50].

## Results

**Figure 2:**
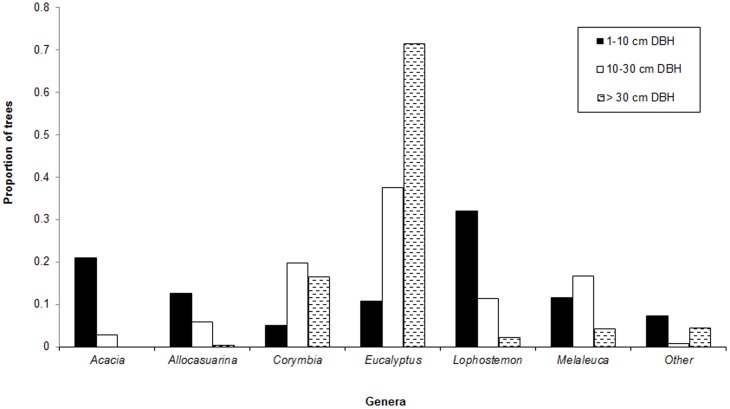
Proportion of trees in selected genera for each cohort at Karawatha.

**Figure 3:**
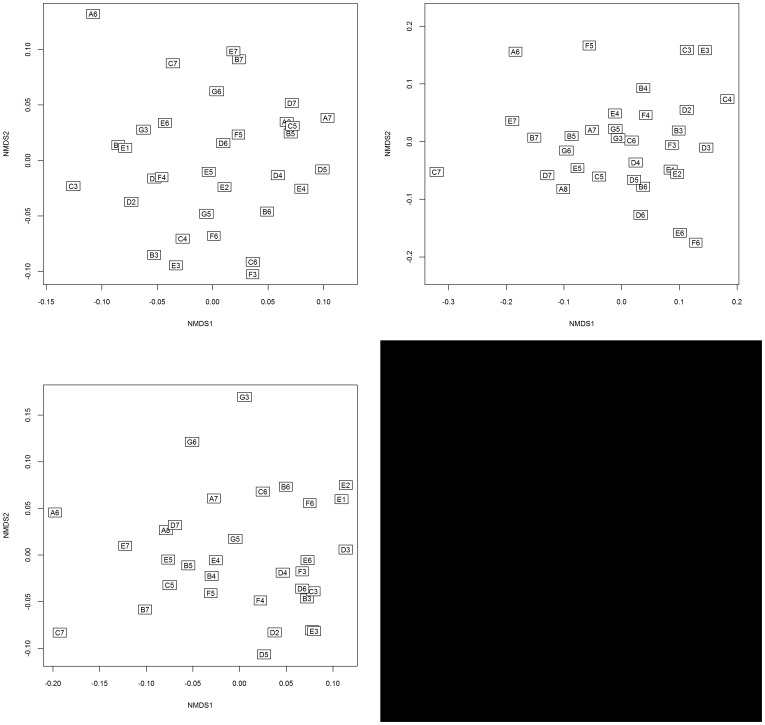
NMDS ordination for: woody vegetation with a DBH 1–10 cm (top-left); woody vegetation with a DBH 10–30 cm (top-right); and c.) woody vegetation with a DBH>30 cm (bottom-left). Although three NMDS axes were used, only the first two are shown for visual representation.

Overall, 49 woody species were identified from 10 825 sampled individuals. The most common genus was *Eucalyptus*, which made up 33% of the coverall composition, while *Lophostemon*, *Corymbia*, *Melaleuca* and *Acacia* were also well represented genera (Table 1). Species composition differed between cohorts, with species diversity being the most even within the youngest cohort (Table 1, Figs. 2 and 3). As the DBH of the cohorts increased, the proportion of trees that were eucalypts also increased.

**Table 1:** The total species richness of each genus within each size cohort among the 33 plots at KFP.

Genus richness	1–10 cm DBH	10–30 cm DBH	>30 cm DBH	Total richness	Abundance
*Acacia*	6	4	1	6	1008
*Alectryon*	1	1	0	1	4
*Allocasuarina*	1	1	1	1	810
*Alphitonia*	1	1	0	1	154
*Angophora*	1	2	1	2	459
*Banksia*	1	1	0	1	29
*Callistemon*	1	1	1	1	5
*Corymbia*	5	5	5	5	1456
*Eucalyptus*	18	19	19	21	3620
*Exocarpus*	1	0	0	1	21
*Glochidion*	1	0	0	1	1
*Leptospermum*	1	0	0	1	5
*Lophostemon*	2	2	2	2	1908
*Melaleuca*	4	4	3	4	1344
*Persoonia*	1	0	0	1	1
**Total**	**45**	**41**	**35**	**49**	**10 825**

Plant abundance for each genus is also shown.

### Spatial correlation

There was no significant spatial autocorrelation of woody vegetation composition over a distance 500 m to 2000 m (*r_M_* 0.08–−0.09). However, there was significant dispersion of trees with a DBH of 10–30 cm and>30 cm DBH at a distance of 2000 m among plots (*r_M_* = −0.08 and −0.09, respectively. *P* = 0.03 and 0.02, respectively).

### Patterns of Vegetation Assemblages along Environmental Gradients

Three-dimensional NMDS with a stress of 0.18, 0.15 and 0.15 (small to largest cohorts, respectively) represented the input matrix of vegetation composition and described similarity between plots for all cohorts (Fig. 3). Three soil PCA axis were used for the surface analysis, with Axis I, II and III explaining 45%, 13% and 12% of the variation of the soil correlation matrix, respectively. Axis I was negatively related to Mg^2+^, Na^+^, EC, CEC, C and N (Table 2). The second component was positively related to Ca^2+^ and P and negatively related to K^+^ and Na^+^. Axis III was highly positively associated with NO_3_^−^, positively related to NO_2_^−^ and negatively related to the proportion of silt and pH.

**Table 2:** Correlations of 14 soil variables with the three major axes of the soil PCA analysis.

Variable	Axis I	Axis II	Axis III
% clay	−0.237	0.231	−0.118
% silt	−0.011	0.209	−0.491
pH	−0.097	−0.137	−0.440
EC	−0.330	0.154	0.172
Ca^2+^	−0.269	0.406	−0.095
K^+^	−0.228	−0.525	0.035
Mg^2+^	−0.342	−0.224	−0.054
Na^+^	−0.333	−0.322	0.035
CEC	−0.378	0.062	−0.067
Total P	−0.266	0.428	−0.034
Total C	−0.327	−0.176	0.092
Total N	−0.380	0.012	0.055
NO_2_^−^	−0.087	0.185	0.343
NO_3_^−^	0.005	0.115	0.610
Variation explained (%)	45	13	12

**Figure 4:**
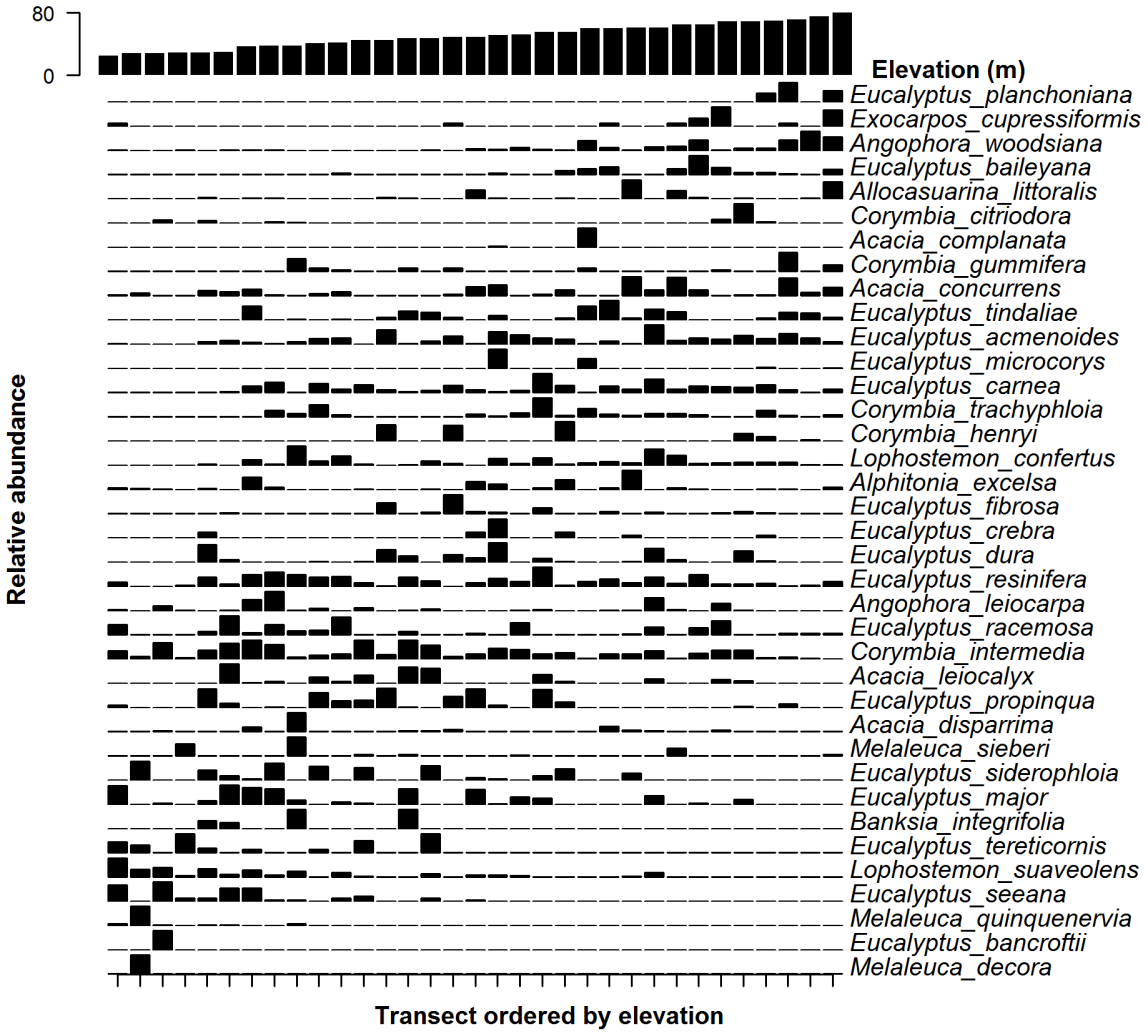
Relative abundance of woody vegetation species ordered by elevation.

Surface modelling indicated that elevation, catchment area, and slope significantly influenced species composition of all cohorts (Table 3). All of these variables can be classed as topographic variables. Elevation explained the most variation in species composition among all cohorts, as demonstrated by the changing composition and relative abundance of species along the elevation gradient (Fig. 4). Soil Axis I was associated with composition in the smallest and largest cohorts (Table 3).

**Table 3:** Surface modelling statistics for each cohort and significant environmental variable.

Variable	1–10 cm DBH *R^2^* Value	10–30 cm DBH *R^2^*	>30 cm DBH *R^2^*
Elevation	0.69***	0.75***	0.69***
Slope	0.23**	0.61***	0.52***
CA	0.43***	0.49***	0.45***
Soil Axis I	0.31*	0.09 NS	0.49***

R^2^ value and P-value are shown. CA is catchment area.

*, *P*<0.05;

**, *P*<0.01;

***, *P*<0.001; NS is *P*>0.05.

## Discussion

This study has shown that variation in tree assemblages in a subtropical eucalypt forest at the mesoscale is strongly related to topographic variables and secondarily related to soil properties. Elevation clearly explained the most variation of composition in all cohorts. The composition of size cohorts was different; however, the strength of environmental variables explaining composition only differed moderately. Composition of all cohorts was not spatially correlated between plots.

### Patterns of Vegetation Assemblages along Environmental Gradients

Elevation has been shown to drive vegetation patterns including functional traits, species richness, species composition and density in numerous studies at a number of spatial scales [51], [52], [53], [54], [55], [56], [57], [58] and in a number of vegetation types [27], [59]. Elevation as an environmental correlate however, has no direct physiological impact on vegetation patterns [60], [61] and acts as a surrogate for the influence of direct factors including catchment flows, slope and soil chemistry (Table 4). A range of environmental variables including hydrology and soil chemistry have also been shown by other studies to vary along narrow elevation gradients [16], [20], [60], [61], [62], [63]. The strong association between a narrow elevation gradient and vegetation patterns as found in this study reflect the role of numerous direct environmental factors [61].

**Table 4:** Relationship between environmental predictor variables.

	Elevation	Aspect	Catchment Area	Slope	Soil Axis I	Soil Axis II
Elevation						
Aspect	−0.23					
Catchment Area	−0.63	0.29				
Slope	0.69	0.00	−0.43			
Soil Axis I	0.30	−0.31	−0.46	0.05		
Soil Axis II	−0.12	0.11	0.10	−0.07	0.00	
Soil Axis III	0.21	−0.17	−0.28	0.17	0.00	0.00

Pearson's correlation coefficients are shown.

Catchment area can influence vegetation patterns because small catchment area represents a relatively smaller amount of available water compared to large catchment areas, especially at the lower areas of the catchment. The influence of water availability including catchment runoff [64] and regional hydrological patterns [64], [65] on vegetation assemblage development has been shown to be closely linked to species habitat requirements. Species with similar hydrological requirements are consequently clustered in optimal areas within catchment systems, resulting in specific assemblage patterns.

Like elevation, slope can indirectly influence habitat suitability and thereby composition at local scales [20], [66] by changing microclimate, runoff, drainage, soil erosion and formation [57], [67]. Although not always the case [16], it appears slope can be a strong driver of species composition that can influence over small [57], [68], meso [15] and large scales [67], [69]. Slope was an important driver in KFP for all cohorts, but more so for larger cohorts.

Although topography influences soil characteristics and thereby drives tree composition, soil chemistry, as measured in this study, generally did not describe tree composition variability as much as topography at KFP. This reflects the findings of Catterall et al. [27], who found that elevation, not measured soil properties, was important in explaining eucalypt composition in a nearby forest patch to KFP. In contrast, Wardell-Johnson et al. [70] showed that soil chemistry was associated with vegetation composition in the same study area used by Catterall et al. [27]. Different sampling regimes were used between the two studies, with coverage and the number of replicates of Wardell-Johnson et al. [70] being comparatively higher. On a broad scale, Wills and Clarke [71] summarised that Australian vegetation types change with rainfall, temperature and soil. Further afield, Pansonato et al. [72] summarised that many studies in western Amazon have found that soil fertility was an important driver of plant communities, while central Amazonian studies have found that topography or soil texture were predictors of vegetation communities. Further studies have also found that topography and soil influence vegetation composition at small [68] and large scales [4] and Eiserhardt et al. [3] suggested that climate was more of a driver of palm vegetation patterns at broader scales, but topography and soil influenced vegetation at smaller scales. Considering there is some, but not complete correlation between soil and topography, it is not surprising that both soil chemistry and topography influenced different cohorts within KFP at the mesoscale. Pansonato et al. [72] used PPBio plots in Brasil to show that it was the amplitude of environmental factors and not the scale of these factors that was important in explaining composition. While the elevation only ranged from 28–80 m at KFP, the soil types did vary from well-drained, infertile sandstone on the ridges to richer alluvial soil in the wetlands. Similarly, this variation was also associated with the turnover of the woody vegetation community at KFP (Fig. 4).

The gradient of vegetation from ridge tops to valley floors detected here (Fig. 4) is consistent with similar studies on a variety of vegetation types and spatial scales [17], [20], [27], [55]. This model is characterised by the inherent differences between valley floors and ridge tops, where valley floors have relatively higher soil moisture, a shallower water table, and higher nutrient loading compared to upslope and ridge top areas [17], [20], [27]. These studies however, did not sample over the entire catchment gradient, instead only sampling at the tops of ridges and at the bottom of valley floors which provides an easily detected difference. Our study therefore contributes to an important, although generally only assumed, body of literature on the gradient of tree assemblages driven by water and nutrient availability.

The eucalypt assemblages at Karawatha were distributed along a gradient of changing tree species abundance without clear boundaries. This spatial pattern is in contrast to the community-unit theory where plant communities form homogenous, distinct and identifiable units [1]. However, the inherent difference between observing vegetation patterns as communities or as changing assemblages along a continuum depends on the spatial and temporal scale of the study [12], [73]. For example, Battaglia and Williams [12] demonstrated that eucalypt savannas changed along a continuum if viewed at the soil nutrient scale, although clear community boundaries were present if viewed at the soil type scale. This demonstrates that at the mesoscale, catchment flow and soil gradients may be too narrow to produced identifiable vegetation communities due to the lack of distinct environmental habitats along the gradient from ridge-tops to riparian areas, although large enough to detect significant change in species assemblages across the catchment gradient.

### Differences between Vegetation Size Cohorts

While elevation was one of the best environmental determinants of tree assemblages, there were slight variations in the relative importance of other environmental variables for the three size cohorts (Table 4). Slope explained more variation in the two larger cohorts, soil was not a significant predictor in the middle cohort and catchment area was a similarly important driver within all cohorts. While these differences may reflect the role of selective pressures on the abundance of trees during different stages of their life history [31] the differences detected in our models are relatively small. This suggests that the selective pressures of species abundance patterns in subtropical eucalypt forest may be relatively constant through each life cycle phase.

Despite little difference in the environmental determinants there were substantial differences in species composition and abundance within the three size cohorts (Figs. 2 and 3). The dissimilarity between size cohorts appears to be related to differences in the life history strategies of species dominating each size cohort. Pioneer species and species restricted to the understorey such as *Lophostemon* spp., *Acacia* spp. and *Allocasuarina littoralis* were relatively common in the smaller cohort, but became less common as cohort size increased. Intermediate and large trees from the genera *Eucalyptus* and *Corymbia* dominated the larger size cohorts. These differences reflect the turnover or succession of species through each consecutive size cohort [74]. Fire may also shape the vegetation composition and structure by killing a disproportionate number of trees in the smallest cohort [75]. The structural and compositional differences between size cohorts therefore reflect the interaction between tree species life history strategies and environmental processes.

### Future Directions and Management Implications

Studying plant physiology at KFP would likely shed more light on the mechanisms behind species turnover. This is because certain physiological characteristics such as leaf mass, stem height and density, root depth and seed size are likely to favour certain environmental conditions and therefore differ between species [76], including between eucalypt species [77]. Less than 300 km away from KFP, Kooyman et al. [58] showed that tree height, wood density, leaf size and seed size was related to elevation in rainforest vegetation. Pollock et al. [78] showed that seed size in eucalypts in Victoria was related to topography and soil type, while Wills and Clarke [71] showed that trait types were associated with soil fertility.

Sampling in this study used PPBio for the purpose of long-term monitoring, therefore resampling of vegetation data will reoccur. This will give insight into vegetation change over time in relation to environmental variables, as demonstrated by [79] and [80]. Studying changes in tree cohort structure, species turnover and mortality may show that tree age structure, composition and abundance are related to dynamic variables such as weather patterns.

This study has shown that in a subtropical eucalypt forest patch, the tree assemblage patterns changed as a continuum along topographic and soil gradients at the mesoscale. Past research together with this study demonstrate that vegetation conservation at the assemblage level needs to consider the role of ecological processes at a variety of spatial scales. Mesoscale ecological studies also need to be replicated at a regional level to take into account the large distribution patterns of many vegetation assemblages and individual species. This would account for local and regional ecological effects and increase the power with which to elucidate the drivers of mesoscale vegetation patterns.
